# Early detection of pine wilt disease based on UAV reconstructed hyperspectral image

**DOI:** 10.3389/fpls.2024.1453761

**Published:** 2024-11-19

**Authors:** Wentao Liu, Ziran Xie, Jun Du, Yuanhang Li, Yongbing Long, Yubin Lan, Tianyi Liu, Si Sun, Jing Zhao

**Affiliations:** ^1^ College of Electronic Engineering (College of Artificial Intelligence), South China Agricultural University, Guangzhou, China; ^2^ College of Forestry and Landscape Architecture, South China Agricultural University, Guangzhou, China; ^3^ National Center for International Collaboration Research on Precision Agricultural Aviation Pesticides Spraying Technology, Guangzhou, China

**Keywords:** pine wilt disease (PWD), early stage detection, UAV remote sensing, hyperspectral image reconstruction, supervised classification

## Abstract

Pine wilt disease (PWD) is a highly destructive infectious disease that affects pine forests. Therefore, an accurate and effective method to monitor PWD infection is crucial. However, the majority of existing technologies can detect PWD only in the later stages. To curb the spread of PWD, it is imperative to develop an efficient method for early detection. We presented an early stage detection method for PWD utilizing UAV remote sensing, hyperspectral image reconstruction, and SVM classification. Initially, employ UAV to capture RGB remote sensing images of pine forests, followed by labeling infected plants using these images. Hyperspectral reconstruction networks, including HSCNN+, HRNet, MST++, and a self-built DW3D network, were employed to reconstruct the RGB images obtained from remote sensing. This resulted in hyperspectral images in the 400-700nm range, which were used as the dataset of early PWD detection in pine forests. Spectral reflectance curves of infected and uninfected plants were extracted. SVM algorithms with various kernel functions were then employed to detect early pine wilt disease. The results showed that using SVM for early detection of PWD infection based on reconstructed hyperspectral images achieved the highest accuracy, enabling the detection of PWD in its early stage. Among the experiments, MST++, DW3D, HRNet, and HSCNN+ were combined with Poly kernel SVM performed the best in terms of cross-validation accuracy, achieving 0.77, 0.74, 0.71, and 0.70, respectively. Regarding the reconstruction network parameters, the DW3D network had only 0.61M parameters, significantly lower than the MST++ network, which had the highest reconstruction accuracy with 1.6M parameters. The accuracy was improved by 27% compared to the detection results obtained using RGB images. This paper demonstrated that the hyperspectral reconstruction-poly SVM model could effectively detect the Early stage of PWD. In comparison to UAV hyperspectral remote sensing methods, the proposed method in this article offers a same precision, but a higher operational efficiency and cost-effectiveness. It also enables the detection of PWD at an earlier stage compared to RGB remote sensing, yielding more accurate and reliable results.

## 1 Introduction

Pine wilt disease (PWD) is a highly destructive conifer disease with a global impact, affecting numerous countries and regions. PWD initially originated in North America (Ikegami and Jenkins, 2018) but has now spread extensively across East Asia, resulting in significant damage to billions of pine trees and substantial economic and ecological losses (Zhao et al., 2020a; Hao et al., 2022). Therefore, timely detection of PWD is crucial as it enables us to comprehend the current infection situation and implement appropriate measures to prevent further spread. The existing classification of PWD disease cycles primarily defines infection stages based on resin secretion, growth vigor, and needle color (Yu, R et al., 2021a; 2021b). The traditional and widely used method for PWD detection involves analyzing wood samples collected from trees in the chemistry laboratory to detect the presence of pine wood nematode (PWN) (Futai, 2013). However, this method necessitates expertise in nematology and is time-consuming. Additionally, collecting samples over large areas becomes challenging due to the complex terrain of mountainous regions. Thus, there is an urgent need to develop a rapid, non-destructive, and large-scale early detection method.

Recently, UAV-based remote sensing has emerged as a promising approach for detecting forest pests and diseases (Yang et al., 2017; Xiao et al., 2022; Du et al., 2024). The integration of UAV-based remote sensing in plant disease detection has garnered extensive acceptance due to its cost-effectiveness, flexibility, and superior spatial resolution, surpassing conventional manual detection methods (Bagheri, 2020; Sangaiah et al., 2024; Shan et al., 2024; Zhao et al., 2023; Guo et al., 2023). On the other hand, a large number of studies show that there are differences in the visial-infrared spectral band of the canopy before and after PWD infection (Kim et al., 2018; Yu et al., 2021a). Based on this fact, researchers have begun using the method of spectral method to do the PWD detection (Li et al., 2022; Yu, R et al., 2021b; Rao et al., 2023; Oide et al., 2022), the U-shaped network architecture, known for its efficacy in capturing features across varied scales of image space dimensions, is adopted. (Zeng et al., 2021). So to combine these two methods together has become a hotspot in PWD research. Certain research methods integrating UAV and spectral images with target detection algorithms enables direct image-based detection (Wu et al., 2021; Yu et al., 2021c). Take Wu, B and Yu, R., for example. Wu, B et al. trained four deep learning models for detection purposes, which utilized collected images in the 390-760nm range to detect Early stage PWD, resulting in an average detection accuracy of 50.2%. Yu, R., et al. used the random forest based on cart decision tree model to detect PWD early infection stage, which extracted 37 spectral variables from hyperspectral data and the recognition accuracy reached 71.67%. Nevertheless, the mainstream UAV hyperspectral cameras operate on push-broom imaging principles, compromising spatial resolution in favor of spectral information, which leads to low image acquisition efficiency and strict demands on stable environmental illumination. This limitation hampers their suitability for outdoor environments and large-area detection. Conversely, the advancement of deep learning technology has notably enhanced the performance of hyperspectral image reconstruction networks. A viable approach involves utilizing UAV RGB cameras to capture color remote sensing images and conduct hyperspectral image reconstruction, enabling the extraction of spectral information for disease testing purposes.

Currently, hyperspectral images reconstruction networks can accurately retrieve spectral information from RGB images. The accuracy of hyperspectral reconstruction has been consistently enhanced in recent computer vision summit challenges, including NTIRE2018 (Arad et al., 2018), NTIRE2020 (Arad et al., 2020), and NTIRE2022 (Arad et al., 2022). Among them, the NTIRE2018 champion network HSCNN+ is a convolutional neural network-based method for hyperspectral image reconstruction (Shi et al., 2018). In the NTIRE2020 champion network HRNET, a 4-level hierarchical regression network method is used to reconstruct hyperspectral images (Zhao et al., 2020b). The network utilizes PixelUnShuffle and Pixelshuffle layers for inter-layer downsampling and upsampling (Shi et al., 2016), thereby preserving more spectral details. In NTIRE2022, the champion network MST++ employed the Transformer (Ashish et al., 2017) as the fundamental framework for hyperspectral image reconstruction (Cai et al., 2022). To enhance the performance of spectral reconstruction, particularly for reconstructing UAV remote sensing hyperspectral images in large-scale forest monitoring, this study introduces a novel spectral reconstruction network called DW3D. DW3D employs depth-separable three-dimensional convolution to minimize the network parameter count (Rahim et al., 2021) and utilizes a U-shaped network architecture to extract features at various scales (Ronneberger et al., 2015), resulting in improved reconstruction performance. This network architecture exhibits strong expressive and generalization abilities in spectral reconstruction tasks. This article employs the three champion reconstruction networks and the self-built DW3D network architecture to conduct spectral reconstruction on RGB images captured by UAV remote sensing, specifically for the task of early PWD detection.

Therefore, we presented a novel method for early detection of PWD, specifically based on UAV remote sensing hyperspectral image reconstruction and SVM classification. The contributions of this article can be summarized as follows:

1. This study combined the hyperspectral image reconstruction method with an SVM classification model to achieve Early stage detection of PWD using UAV RGB remote sensing images.
2. This paper proposed a novel hyperspectral reconstruction network that combines three-dimensional depth-separable convolution and a U-shaped network to enhance detection efficiency and enable real-time detection of UAV remote sensing over large areas.
3. Establish a UAV hyperspectral PWD image dataset containing healthy pine trees and pine trees in the Early stages of PWD.

## 2 Materials

**Figure 1:**
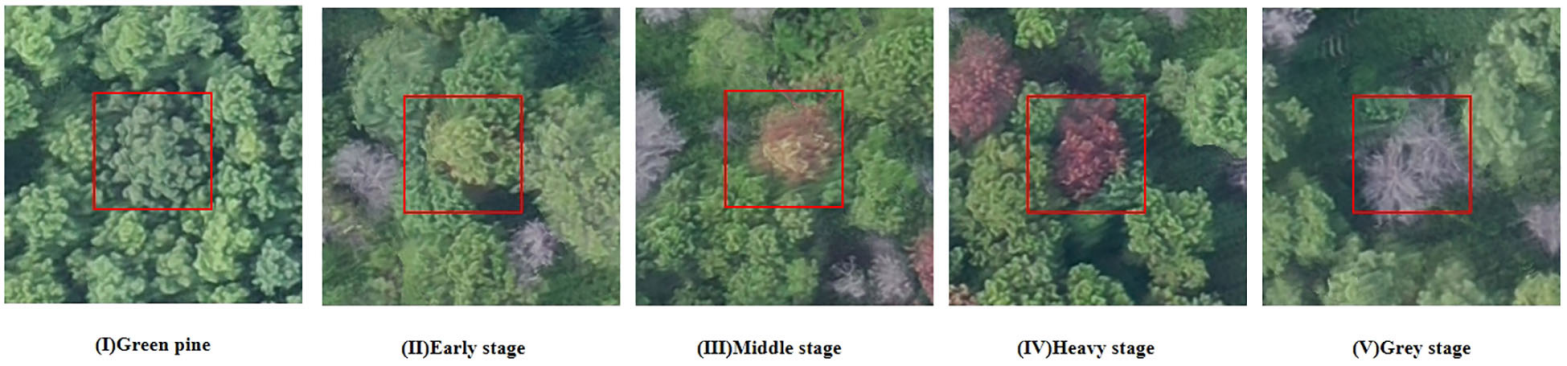
Unmanned Aerial Vehicle (UAV) images of pine trees at different PWD infection stages.

This paper utilized two datasets: a self-built dataset for detecting PWD and a public dataset for training the hyperspectral image reconstruction network. Furthermore, by combining needle analysis, ground plant observations, and drone images, PWD is categorized into five stages: (I) Green pine, (II) Early stage, (III) Middle stage, (IV) Heavy stage, and (V) Gray stage (Yu, R et al., 2021). The remote sensing images provided by this article are also divided into 5 categories, as depicted in **Figure 1**.

### 2.1 The self-built dataset of pine forest hyperspectral images

**Figure 2:**
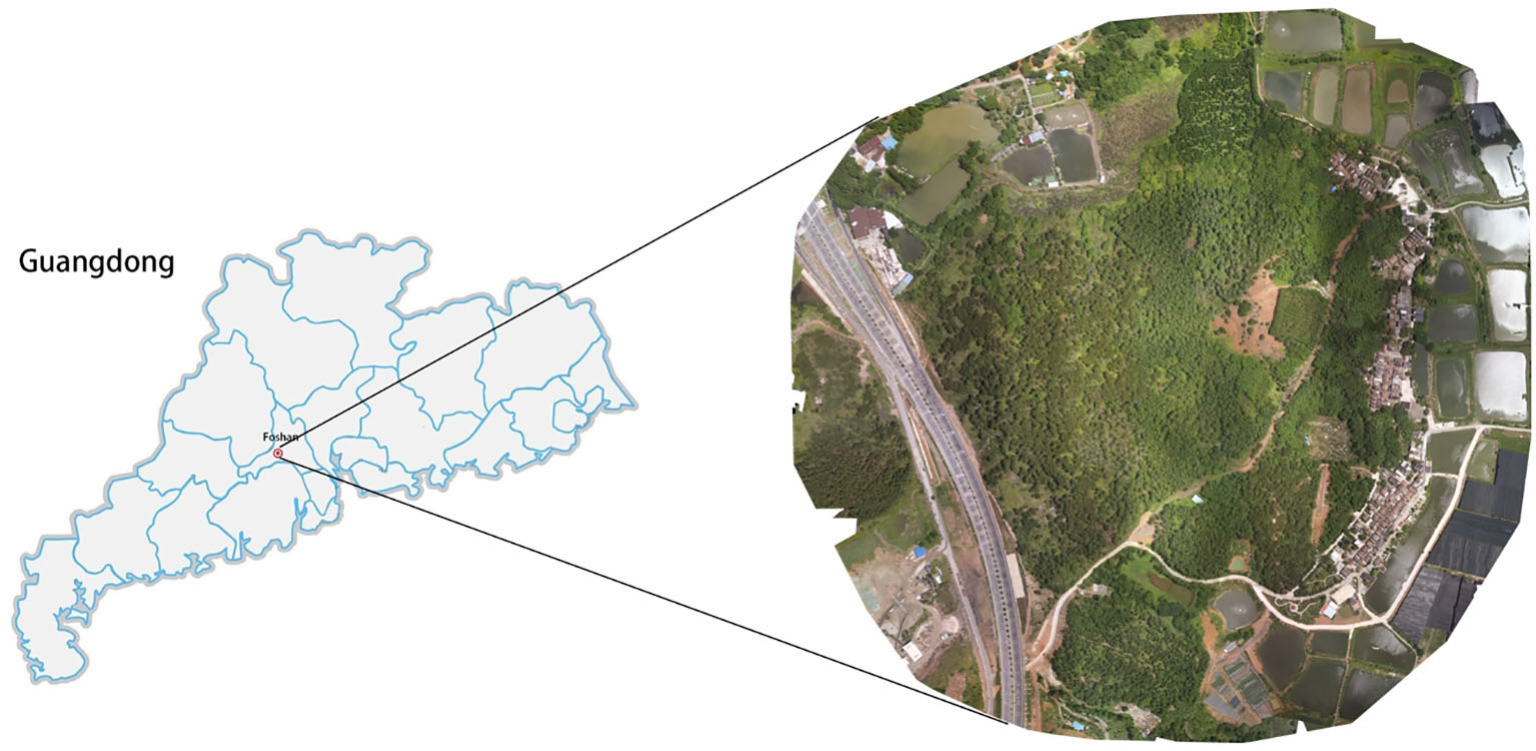
A stitched image of the study area.

The pine remote sensing dataset utilized in this experiment was sourced from a forest farm located in Hecheng Street, Gaoming District, Foshan City, Guangdong Province, China(22°54′N, 112°51′E). The forest farm spans 57 hectares and predominantly consists of masson pine trees. The dataset comprises UAV remote sensing images captured at the same location over a duration of eight months, from May to December 2022. Data collection was performed using the DJI Phantom 4RTK drone, with each flight carried out at an altitude of 350 meters. Data collection took place in sunny weather conditions between 10:00 and 14:00, with a light intensity of approximately 100,000 Lux, to maintain data collection consistency. The RGB camera had a spatial resolution of 1600x1300 pixels, as depicted in **Figure 2**.

**Figure 3:**
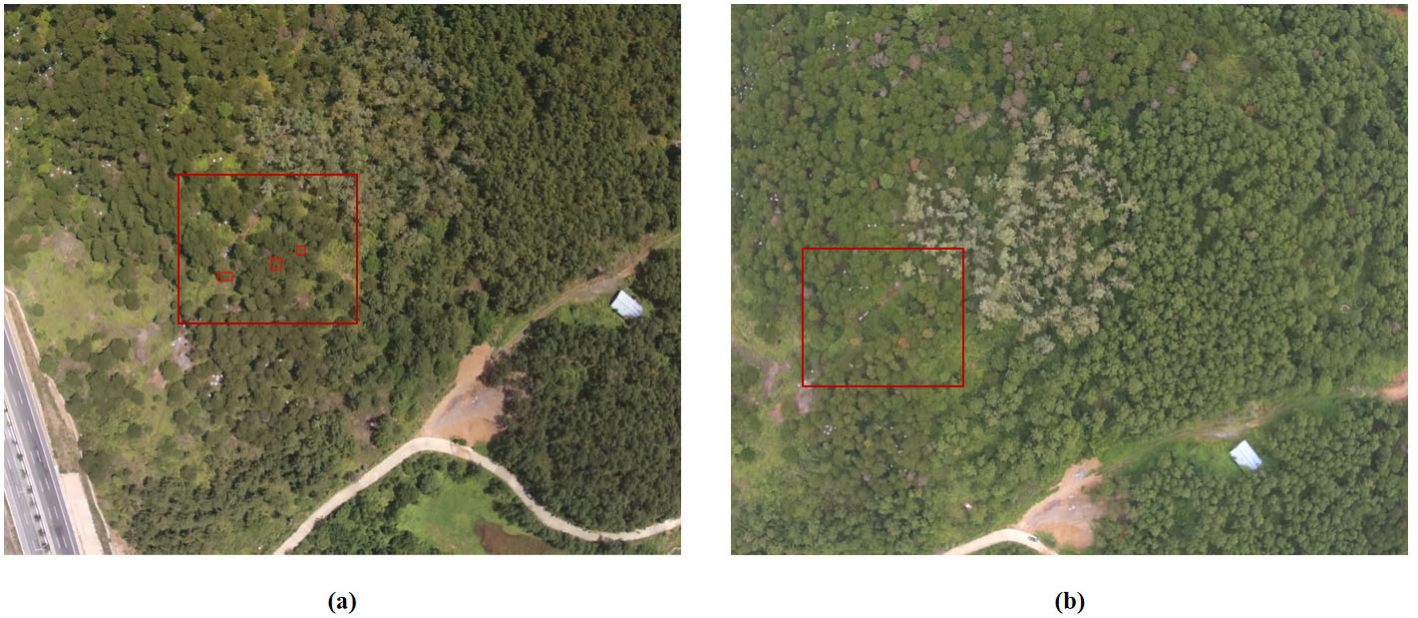
**(A)** Correspond to the images captured on May 26 2022, and **(B)** correspond to the images captured on June 29 2022. Mid to late-infected pine trees in the newer image identifying early-infected pine trees in the older image by coordinates.

This study conducted 10 remote sensing data collections spanning from May to December 2022. The study utilized the coordinates of mid to late-infected pine trees in more recent images to identify early-infected pine trees in earlier images. Additionally, ground inspection confirmed that the target plants were infected with pine wilt disease (PWD) As shown in **Figure 3**.

**Figure 4:**
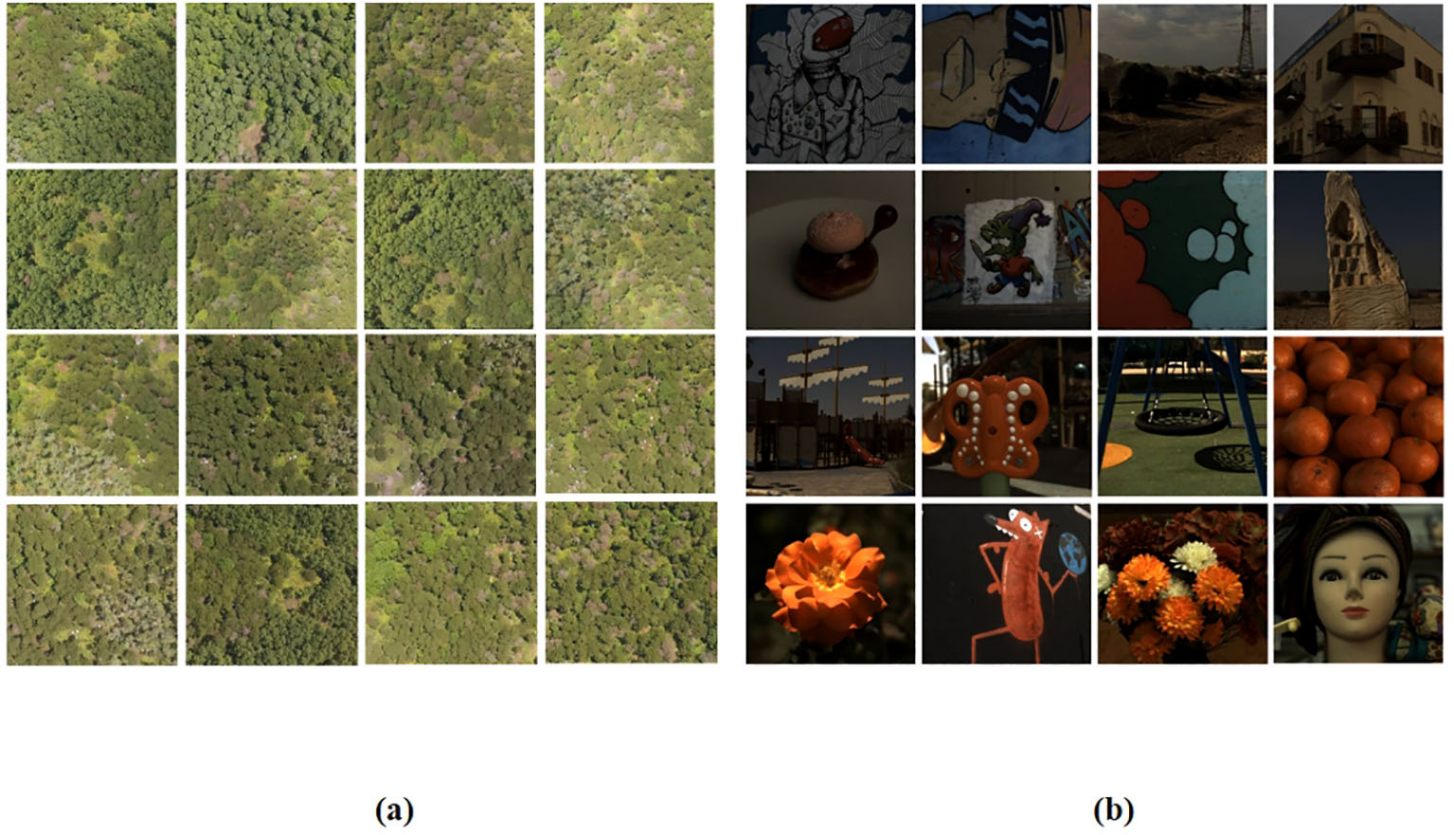
**(A)** UAV RGB remote sensing image dataset, and **(B)** the NTIRE2022 Spectral Reconstruction Challenge open source dataset.

**Figure 5:**
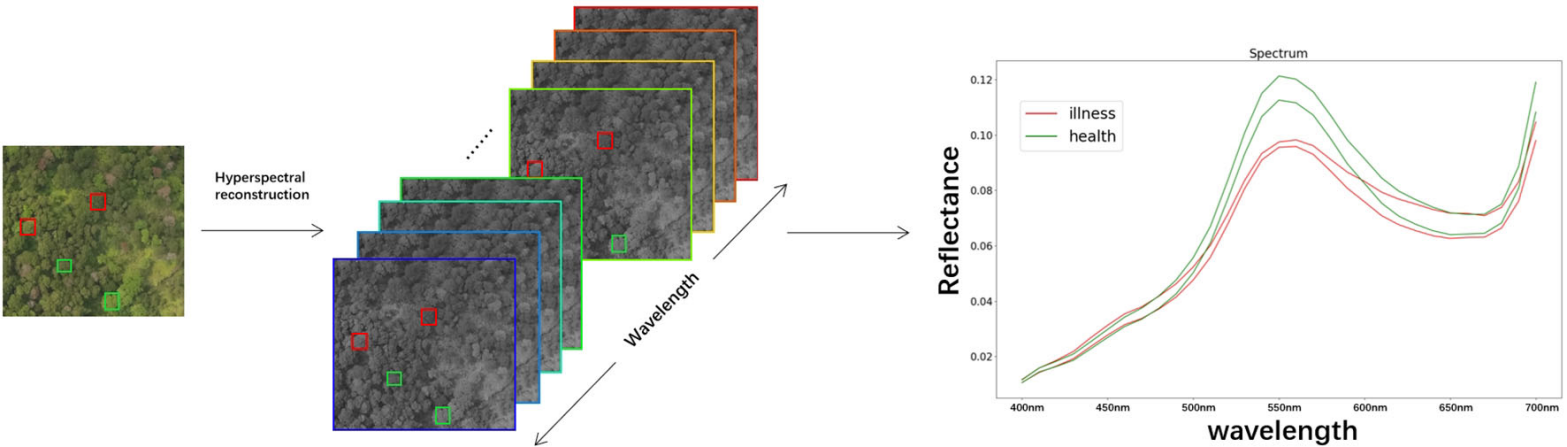
Hyperspectral images reconstruction and reflectance spectral feature acquisition.(The red box represents the diseased plant, the green box represents the healthy plant, and the curve depicts the spectral characteristic curve of the plant.).

Following manual annotation of the original remote sensing images, the UAV remote sensing images were cropped to achieve a resolution of 512x482. As shown in **Figure 4A**. The cropped images were processed by the hyperspectral reconstruction network, resulting in the creation of the corresponding dataset of pine forest hyperspectral images. The spectral information of the corresponding plant is extracted from the reconstructed hyperspectral image based on the annotation. As shown in **Figure 5**.

A total of 320 target plants, consisting of equal proportions of healthy and diseased plants, were obtained based on the annotations. Eventually, a spectrum dataset consisting of 320 samples were obtained, with each sample containing 31 spectral features and a label indicating the presence or absence of disease. A total of 31 spectral features are utilized for machine learning classification.

### 2.2 The public dataset

The NTIRE2022 spectral reconstruction public dataset (Arad et al., 2022) is employed in this article to pre-train the four reconstruction networks utilized in our study. The dataset comprises 1000 RGB-HSI pairs, which are partitioned into training, validation, and test subsets in an 18:1:1 ratio. Each HSI data possesses a spatial resolution of 482 × 512 and encompasses 31 wavelengths ranging from 400 nm to 700 nm, as depicted in **Figure 4B**.

## 3 Methods

Deep networks are employed in this article for the reconstruction of hyperspectral images. The champion networks, HSCNN+, HRNet, and MST++ from the NTIRE2018, NTIRE2020, and NTIRE2022 Spectral Reconstruction Challenges, were utilized. Additionally, the self-built spectral reconstruction network DW3D from this article was employed to reconstruct hyperspectral images of pine forest remote sensing.

Simultaneously, this study employed the SVM classification model to classify and detect Early stage of PWD using the reflectance spectral features of target plants obtained through crown segmentation from the pine forest reconstructed hyperspectral images.

### 3.1 Hyperspectral reconstruction network

#### 3.1.1 DW3D self-built network

DW3D is a hyperspectral reconstruction network that combines three-dimensional convolution and the U-Net architecture. Depthwise separable three-dimensional convolution is employed to reduce the parameter count, contributing to a more efficient network. It achieves improved reconstruction performance by performing multi-scale feature extraction through a U-shaped network architecture.

**Figure 6:**
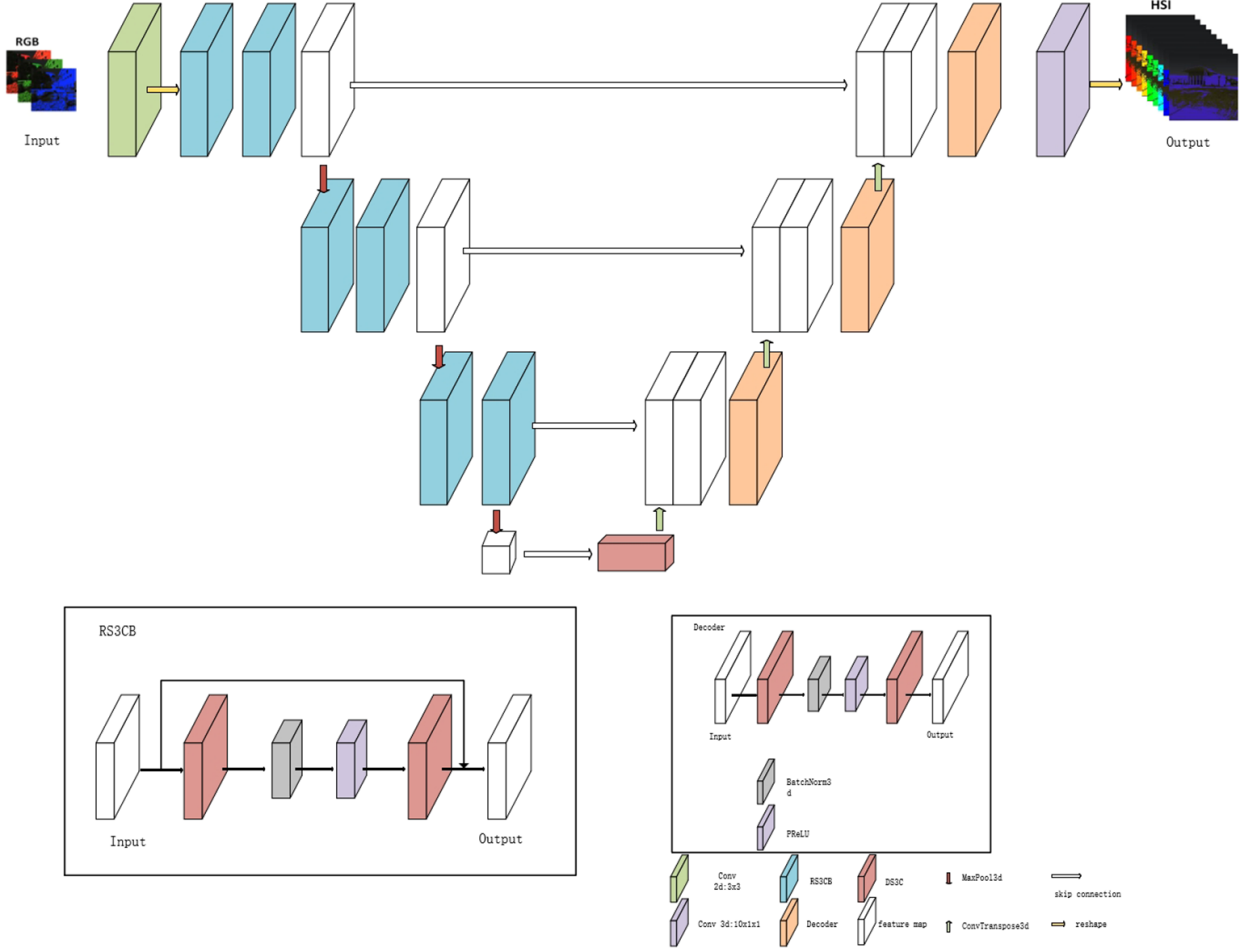
Self-built DW3D structure.

**Figure 6** illustrates that the input of the DW3D network is an RGB image. Initially, the feature channels of the RGB input undergo expansion using a separate two-dimensional convolution layer, followed by a matrix dimension transformation to include a depth dimension. Subsequently, a deep feature extraction module is constructed using three encoder modules and three decoder modules, forming a combination of U-shaped networks. To alleviate the issues of information loss and blurring during network transmission, a skip connection is employed, allowing direct transfer of intermediate features from the encoder module to the decoder module. Residual connections are incorporated in the deep feature extraction module to address the challenges of gradient disappearance and gradient explosion. The encoder module comprises two residual separable 3D convolution blocks (RS3CB), and the decoder module includes two depth-separable 3D convolutions (D3C) (Rahim et al., 2021). To enhance convergence speed and improve the model’s generalization ability, batch normalization and the PReLU activation function are applied. RS3CB is composed of two depthwise separable 3D convolutions and a PReLU activation function. Batch normalization is applied prior to the activation function, and residual connections are utilized within RS3CB. Downsampling in the U-shaped network is performed using a three-dimensional maximum pooling layer, whereas upsampling is achieved using a three-dimensional transposed convolution layer. In contrast to conventional upsampling approaches like nearest neighbor interpolation and bilinear interpolation, the three-dimensional transposed convolution layer is equipped with learnable parameters (Dumoulin and Visin, 2016) and can dynamically adjust the upsampling parameters based on the network. Lastly, the output feature cube from the deep feature extraction module is transformed into a feature cube with 31 channels using a three-dimensional convolution operation. Subsequently, a hyperspectral image comprising 31 channels is obtained by reshaping the matrix dimensions.

**Figure 7:**
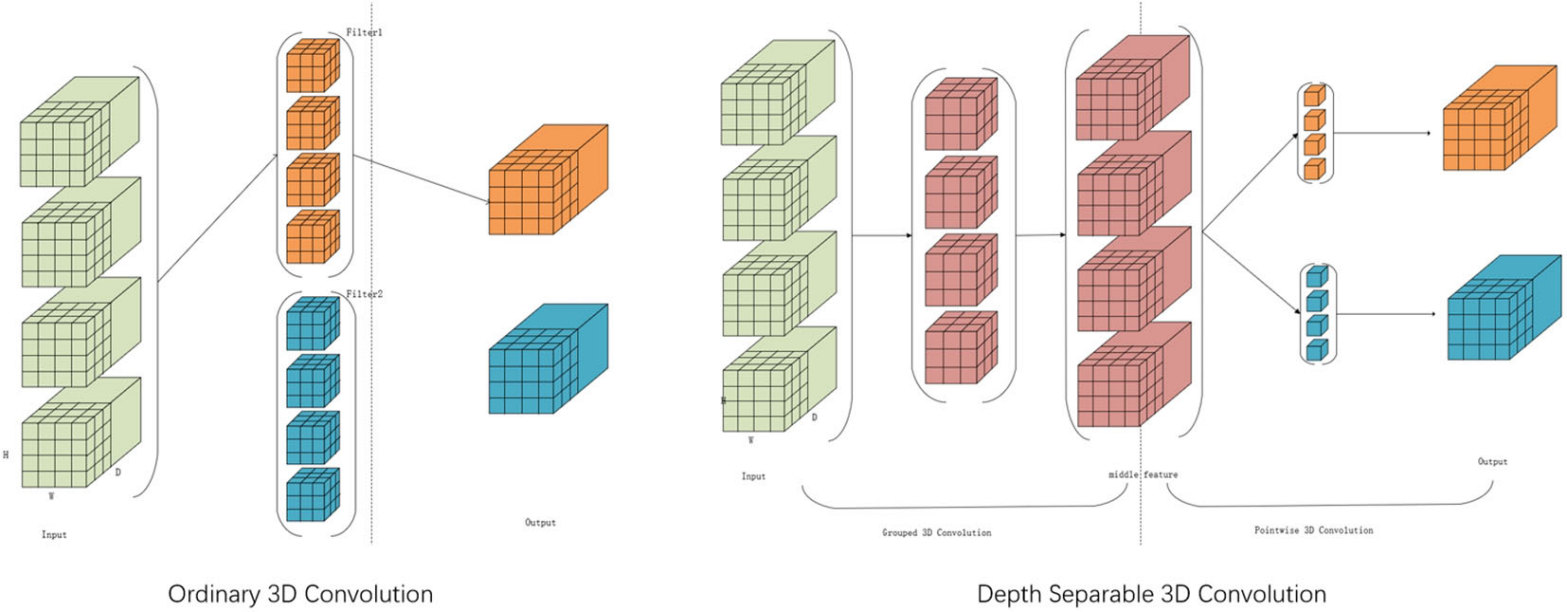
Depthwise Separable 3D Convolutional Module (D3C).

In this study, **Figure 7** illustrates the employment of the Depthwise Separable 3D Convolution Module (D3C). Given that 3D convolution possesses an additional dimension compared to 2D convolution, it offers advantages in channel dimensionality. Concurrently, the U-shaped network architecture, known for its efficacy in capturing features across varied scales of image space dimensions, is adopted. Traditional 3D convolution bases its convolution kernel count on input and output channel numbers, leading to potential parameter redundancy. To address this, the study suggests utilizing Depthwise Separable 3D convolution to trim unnecessary parameters while maintaining the reconstruction quality. This technique segments the convolution layer into two components: a grouped 3D convolution and a pointwise 3D convolution. Initially, kernels with matching input channels extract diverse channel features, followed by one-dimensional kernels for channel information fusion. This approach balances feature extraction and channel information fusion, effectively reducing parameters without compromising reconstruction outcomes.

#### 3.1.2 Hyperspectral reconstruction network evaluation

To assess the performance of DW3D proposed in this article on the NTIRE2022 dataset in an objective manner, the scoring criteria provided by the NTIRE2022 Spectral Reconstruction Challenge are followed. The evaluation of this article employs RMSE(root mean square error), MRAE(mean relative absolute error), and PSNR(Peak signal-to-noise ratio) as indicators. MRAE is selected as the ranking criterion to prevent the introduction of weighting errors in high-brightness areas of the test image, rather than using RMSE. The calculation of MRAE, RMSE and PSNR is as follows:


(1) $$\text{MRAE}=\frac{1}{\text{N}}\sum_{\text{p}=1}^{\text{N}}\left(\frac{\left|{\text{I}}_{\text{HSI}}^{\left(\text{p}\right)}-{\text{I}}_{\text{SR}}^{\left(\text{p}\right)}\right|}{{\text{I}}_{\text{HSI}}^{\left(\text{p}\right)}}\right)$$

(2) $$\text{RMSE}=\sqrt{\frac{1}{\text{N}}\sum_{\text{p}=1}^{\text{N}}{\left({\text{I}}_{\text{HSI}}^{\left(\text{p}\right)}-{\text{I}}_{\text{SR}}^{\left(\text{p}\right)}\right)}^{2}}$$

(3) $$\text{PSNR}=10\cdot {\text{log}}_{10}\left(\frac{{\text{MAX}}^{2}}{\text{MSE}}\right)$$

(4) $$\text{MSE}=\frac{1}{\text{N}}\sum_{\text{p}=1}^{\text{N}}{\left({\text{I}}_{\text{HSI}}^{\left(\text{p}\right)}-{\text{I}}_{\text{SR}}^{\left(\text{p}\right)}\right)}^{2}$$

where ${\text{I}}_{\text{HSI}}^{\left(\text{p}\right)}$ and ${\text{I}}_{\text{SR}}^{\left(\text{p}\right)}$ denote the p-th pixel value of the ground truth and the spectral reconstructed HSI. MAX represents the maximum value among all image points. A smaller MRAE or RMSE indicate better performance.

### 3.2 Machine learning classification model

#### 3.2.1 Classification algorithm

This article employs the support vector machine (SVM) as the classification algorithm (Cortes et al., 1995). It utilizes the soft margin SVM and employs various high-dimensional space mapping methods to transform the spectral data into linearly separable data in high-dimensional space.

Furthermore, this article employs logistic regression. It utilizes decision tree classification and Bayesian classification for detection and compares them with SVM classification results. Based on the outcomes in section 4.2, the study adopts the optimal SVM classification model.

#### 3.2.2 Evaluation

This article utilized the 10-fold cross-validation accuracy (Cross-Validation Accuracy) as the ranking metric to assess the classification results. Additionally, precision, recall, and accuracy were employed to evaluate the detection performance, as shown below.


(5) $$\text{recall}=\frac{\text{TP}}{\text{TP}+\text{FN}}$$

(6) $$\text{precision}=\frac{\text{TP}}{\text{TP}+\text{FP}}$$

(7) $$\text{accuracy}=\frac{\text{TP}+\text{TN}}{\text{TP}+\text{FP}+\text{TN}+\text{FN}}$$

Here, TP(A True Positive) occurs when a positive example is correctly classified as positive, while FN(a False Negative) is recorded when a positive example is erroneously classified as negative. Similarly, TN(a True Negative) is noted when a negative example is correctly classified as negative, and FP(a False Positive) is registered when a negative example is mistakenly classified as positive.

This article expanded the evaluation of classification problems by incorporating ten-fold cross-validation to assess the accuracy and stability of the machine learning model. Ten-fold cross-validation involves the random division of the sample data into 10 parts, with 9 parts selected as the training set in each iteration, and the remaining 1 part used as the test set. After each round, 9 new samples are randomly chosen for training. Following several rounds (fewer than 10), the model and parameters that yield optimal loss function evaluation are selected. This method provides a more comprehensive reflection of the model’s stability and accuracy.

## 4 Results

### 4.1 Training of hyperspectral image reconstruction network

This article compared a self-built hyperspectral reconstruction network with an existing state-of-the-art (SOTA) hyperspectral reconstruction network, including the champion networks HSCNN+ and HRNET from the NTIRE2018 and NTIRE2020 Spectral Reconstruction Challenge, as well as the NTIRE2022’s champion network MST++. The results were presented in **Table 1**. The self-built DW3D reconstruction yields improved results in terms of reconstruction accuracy compared to HSCNN+ and HRNet. However, DW3D exhibited a noticeable gap when compared to MST++. Our self-constructed DW3D network exhibits the lowest number of parameters and computational load. This demonstrates the superior efficiency of our DW3D network. GFLOPS, an acronym for floating-point operations, serves as a metric to assess the computational speed of a model. Typically, a lower GFLOPS value correlates with higher computational speed.

**Table 1:** Comparison of four hyperspectral reconstruction network evaluation indicators.

Methods	Parameter (M)	FLOPS n(G)	MRAE	RMSE	PSNR
HSCNN+	4.65	302.89	0.3814	0.0588	26.36
HRNet	31.70	164.20	0.3476	0.0550	26.89
MST++	1.62	23.10	0.1645	0.0248	34.32
DW3D	0.61	16.42	0.3177	0.0446	28.40

**Figure 8:**
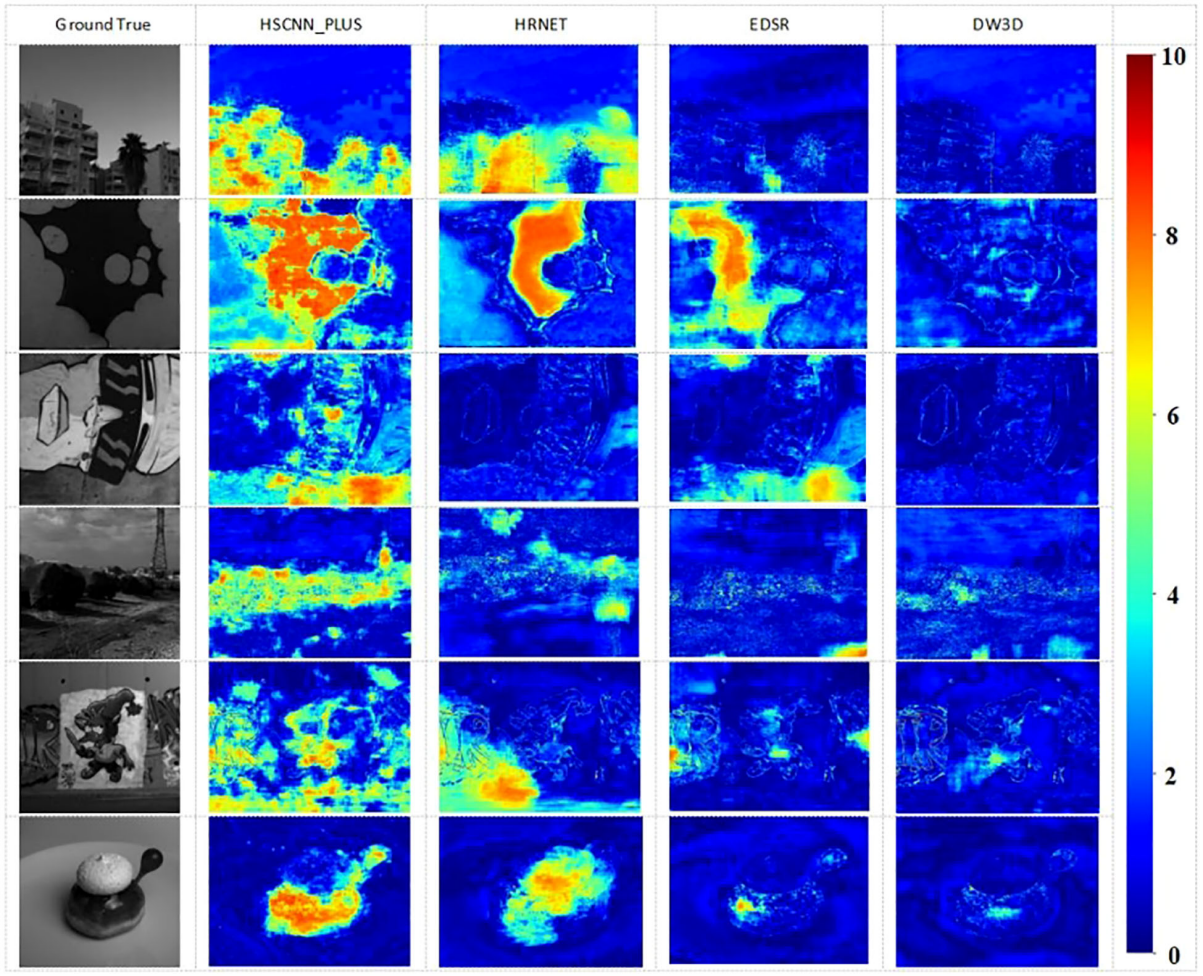
The Visual results of the 21-th band and the reconstruction error images of an HSI chosen from validation set.

This article presented visual HSI reconstruction results and their corresponding error maps for various methods. **Figure 8** displays the error heat map for band 21. Darker colors indicate smaller errors, while lighter colors indicate larger errors. The MRAE error is uniformly distributed across the range from 0 to 10, depicted on the far right of the figure. Based on these figures, it was evident that MST++ exhibits the most favorable reconstruction effect and fidelity, with the DW3D method following closely.

### 4.2 PWD predict results

This study performed conducts a comparative analysis of several different hyperspectral image reconstruction networks combined with SVM classification models for the detection of Early stage PWD. HSCNN+, HRNet, MST++, and the self-built DW3D network were used for hyperspectral image reconstruction and combined with different kernel function SVM classification models, logistic regression classification models, decision tree classification models, and Bayesian classifiers to conduct binary classification experiments. For the classification experiment, the samples underwent shuffling, and a random number seed divided the training and test sets into a 7:3 ratio. The experimental results are shown in **Table 2**.

**Table 2:** Predict results of different reconstruction networks and different kernel SVMs.

Reconstructed Methods	Predicted model	Cross validation	Standard Deviation	Accuracy	Precision	Recall
MST++	Poly-SVM	0.77	0.0744	0.824	0.77	0.82
	Linear-SVM	0.704	0.0774	0.835	0.87	0.80
	Rbf-SVM	0.707	0.0902	0.763	0.77	0.72
	Logistic Regression	0.70	0.0923	0.824	0.71	0.78
	Decision Tree	0.67	0.0855	0.730	0.72	0.80
	Bayes Classifier	0.65	0.0896	0.710	0.70	0.62
DW3D	Poly-SVM	0.74	0.0971	0.793	0.82	0.71
	Linear-SVM	0.669	0.1058	0.8247	0.84	0.66
	Rbf-SVM	0.66	0.0965	0.773	0.86	0.64
	Logistic Regression	0.69	0.1208	0.783	0.78	0.71
	Decision Tree	0.68	0.1032	0.762	0.69	0.73
	Bayes Classifier	0.63	0.1158	0.65	0.71	0.64
HRNet	Poly-SVM	0.719	0.0722	0.763	0.79	0.70
	Linear-SVM	0.70	0.0834	0.793	0.77	0.70
	Rbf-SVM	0.66	0.1003	0.773	0.74	0.68
	Logistic Regression	0.704	0.0856	0.763	0.79	0.70
	Decision Tree	0.64	0.0961	0.793	0.73	0.72
	Bayes Classifier	0.65	0.1143	0.723	0.70	0.68
HSCNN+	Poly-SVM	0.70	0.0658	0.814	0.89	0.70
	Linear-SVM	0.70	0.0657	0.79	0.86	0.77
	Rbf-SVM	0.641	0.1148	0.773	0.78	0.77
	Logistic Regression	0.70	0.1189	0.804	0.83	0.71
	Decision Tree	0.67	0.1139	0.78	0.85	0.77
	Bayes Classifier	0.65	0.1135	0.742	0.62	0.73

Based on the experimental results, the SVM classification model outperforms the other three classification models. We studied the best matching value of the kernel function coefficient (gamma) and the regularization parameter C in the Radial Basis Function (RBF) SVM for PWD data classification in the paper. The gamma value determines the coefficient of the kernel function. The greater the gamma value, the kernel function has a greater impact on the classification results. The experiments demonstrated that the classification cross-validation accuracy was optimal when the kernel function coefficient gamma was set to 0.1 and C was set to 10. This paper investigated the influence of the degree of the polynomial kernel function and the constant term on PWD data classification in polynomial SVM. Modifying the values of degree and constant term alters the model’s capacity to capture nonlinear features. The optimal degree and coef0 are determined by evaluating the cross-validation accuracy. Additionally, the study examined the influence of different regularization parameters (L1 and L2) and various loss functions in the linear kernel function SVM on PWD data classification. Finally, this article employed grid search to identify the optimal parameters by exploring all potential combinations among the candidate parameters. The parameters yielding the best cross-validation accuracy were then selected.

This study ranks using the average accuracy obtained from ten-fold cross-validation and assesses the performance of the classification model by considering standard deviation, accuracy, precision, and recall indicators. **Table 2** reveals the utilization of the self-constructed DW3D network for reconstruction. The poly kernel SVM cross-verification accuracy stands at 74.0%, with the linear kernel SVM achieving the highest accuracy of 82.47%. The MST++ network exhibited a cross-validation accuracy of 77% using the poly kernel SVM, with the highest accuracy of 83.5% achieved using the linear kernel SVM. The results revealed that the self-built hyperspectral reconstruction network outperforms HSCNN+ and HRNet in terms of detection accuracy, generalization, and application performance. It is noteworthy that we achieved a cross-validation accuracy of 74% using our self-built DW3D approach, with the number of model parameters amounting to only 38% of MST++.

**Figure 9:**
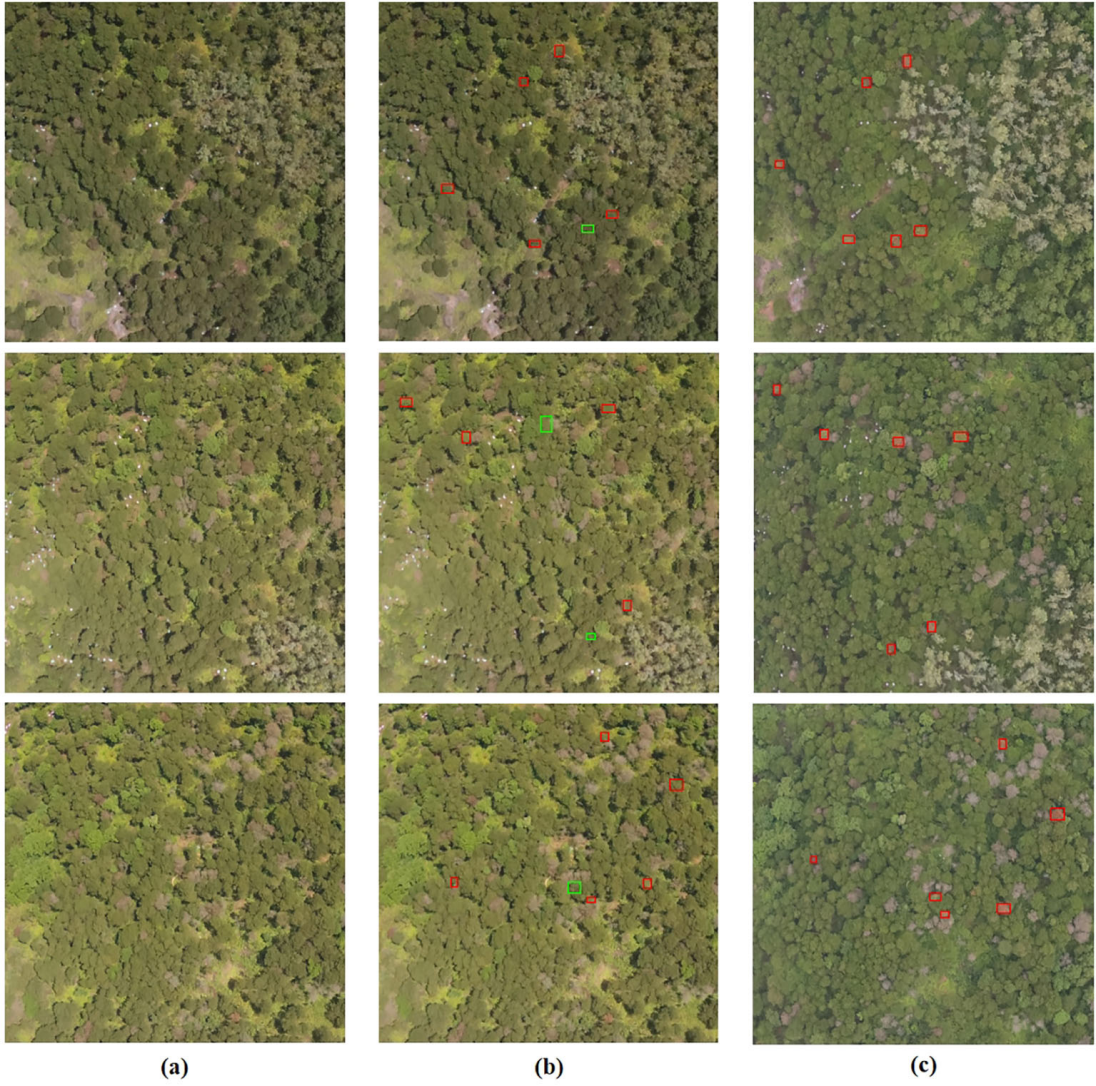
The visualization results of Early stage PWD samples from the test set are presented, with successful classifications denoted in red boxes and incorrect classifications denoted in green boxes. **(A)** represents the original image, while **(B)** represents the predicted result. **(A, B)** Correspond to the images captured on May 26, while **(C)** represents the real image taken on June 29.

In **Figure 9**, This study utilized the MST++-polySVM model to present the visualization results of selected Early stage PWD samples from the test set (the subsequent result analysis defaults to this model). Each sample in the study is assigned a unique number during manual annotation. The annotations of the test set samples are then selected based on their respective test numbers for visual representation. The prediction results are manually annotated on the image based on the plants’ location information, corresponding to the test sample number. Correct classifications are indicated by a red box, while incorrect ones are marked with a green box. In the early PWD detection samples randomly selected, our model can achieve 77% detection accuracy.

This paper currently employs the SVM model to achieve a 77% classification accuracy on a dataset comprising 320 samples, despite having shown promising results in early PWD detection studies. However, there exists ample room for in-depth analysis and enhancement within this study’s findings. Firstly, the quantity and quality of samples play a crucial role. The high flight altitude of the drone in this study results in limited pixels per tree canopy, potentially impacting hyperspectral image reconstruction. Moreover, due to the sample size constraint, the SVM’s performance was not fully realized, potentially influencing the model’s efficacy. Secondly, the selection of model parameters significantly affects accuracy. Experimentation with various kernel functions and parameter combinations has been conducted to optimize detection outcomes. Lastly, feature engineering stands out as a pivotal aspect for enhancing model performance. Future research aims to delve deeper into feature engineering techniques, including feature combination and dimensionality reduction methods, to enhance the model’s data representation capabilities.

## 5 Discussion and conclusion

### 5.1 Advantages of DW3D networks

The self-constructed DW3D model in this paper exhibits substantially fewer parameters and floating-point operations—accounting for only 38% and 71% of MST++, respectively. This indicates that the DW3D model introduced in this study offers accelerated image processing. This makes it feasible to integrate our lightweight reconstruction model into embedded devices, unlike existing reconstruction algorithms that are impeded by their high parameter count. By consuming fewer memory resources and operating with greater speed, our DW3D model enables seamless integration with embedded devices. Moreover, these embedded devices can be incorporated into drone platforms, facilitating rapid, non-destructive, and large-scale detection of early pine wilt disease.

### 5.2 RGB images are directly used for early PWD detection

This paper compared the utilization of RGB images directly, without employing a hyperspectral reconstruction network. The results are presented in **Table 3**. The highest achieved cross-validation accuracy is only 0.50, which means that the use of hyperspectral images is more conducive to early PWD detection.

**Table 3:** Comparison of classification results using RGB color images directly.

Methods	Cross validation	Accuracy
MST++_SVM	0.77	0.824
DW3D_SVM	0.74	0.793
HRNet_SVM	0.719	0.763
HSCNN+_SVM	0.70	0.79
RGB_SVM	0.504	0.531

**Figure 10:**
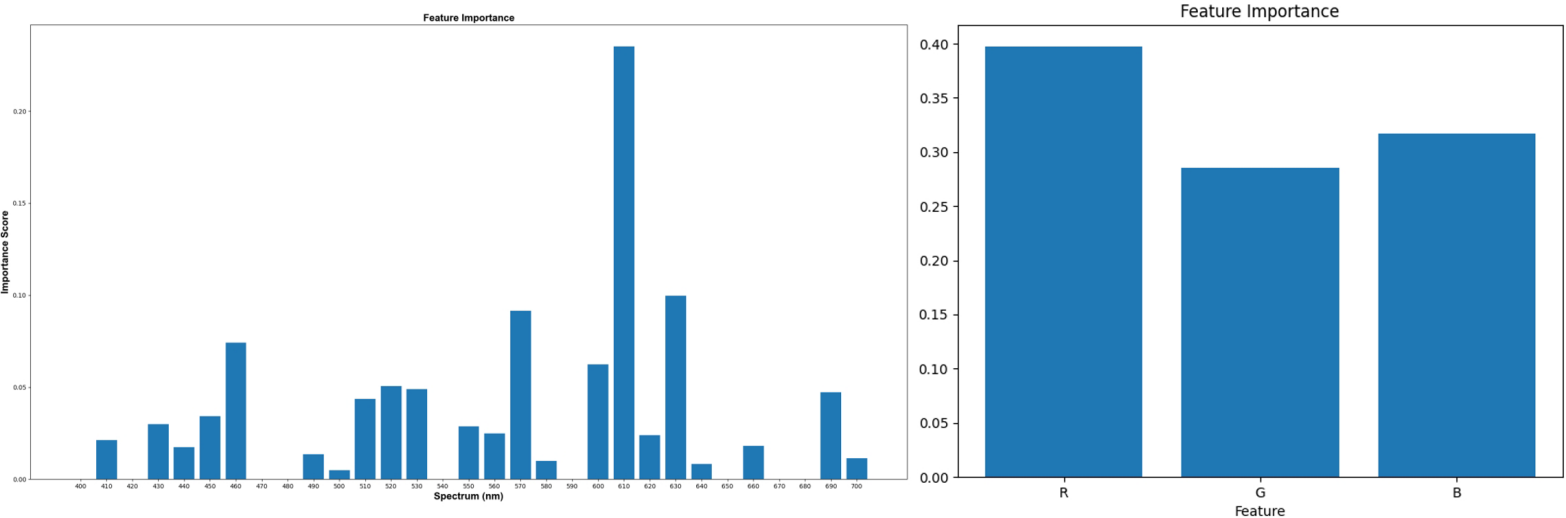
Feature importance of 31 channel features of hyperspectral image (400-700nm, spectral resolution of 10nm) and 3 features of RGB image (R, G, B) to labels.

This study employs a decision tree classification model to assess feature importance. The model evaluates the significance of features by examining their impact on the target variable. Notably, in constructing the decision tree, the optimal partitioning attribute is chosen based on the feature’s classification efficacy on the dataset, utilizing metrics like information gain or information gain ratio. This paper examines and contrasts the contributions of reconstructed hyperspectral images and RGB images in label classification. **Figure 10** displays the ranking of feature importance for labels using 31 features of the reconstructed hyperspectral image and 3 features of the RGB image. The 610nm band exhibits the highest feature importance, succeeded by the 570nm and 630nm bands, as depicted in the figure. The approximate ranges of the three channels in the RGB image are as follows: R (around 700 nm), G (around 550 nm), and B (around 430 nm). The feature importance ranking of hyperspectral images reveals that the features near these channels are of lesser significance. Simultaneously, the feature ranking of RGB images indicates that the importance of the R, G, and B channels is relatively moderate. These three features appear to contain limited classification information, resulting in suboptimal classification performance when using RGB images.

**Figure 11:**
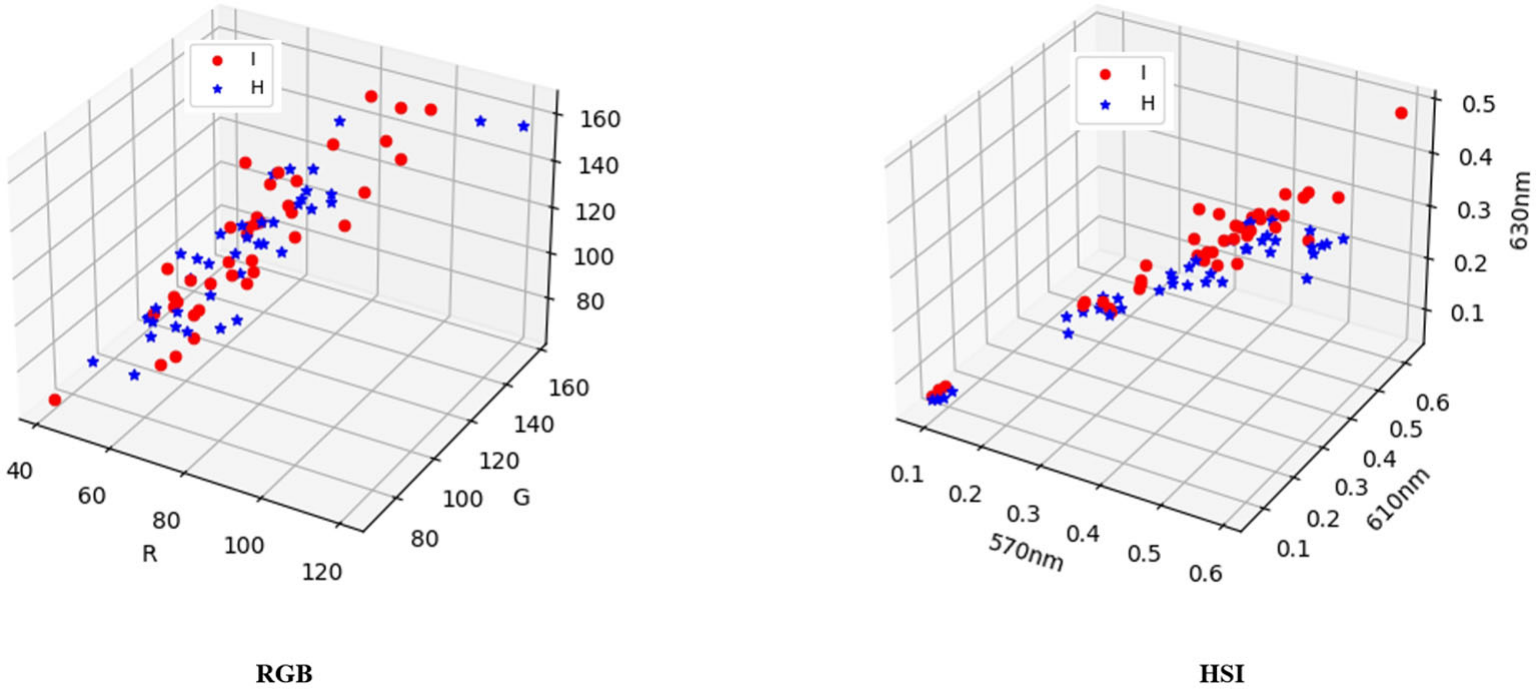
A three-dimensional display of 80 random samples by selecting the three features R, G, and B, and a three-dimensional display of 80 samples by selecting 570nm, 610nm, and 630nm, which have the highest impact on the label.

This article employed a three-dimensional display diagram to compare and analyze the visualization results of three features (R, G, and B) comprising 80 random samples and the channel values (570nm, 610nm, and 630nm) that exert the highest influence on the label. **Figure 11** reveals that the labels in the RGB visualization results lack a distinct classification tendency, whereas the visualization results of the 18th, 22nd, and 24th channels in HSI exhibit a noticeable classification tendency. These findings imply that incorporating features from reconstructed hyperspectral images may enhance the classification performance in this task.

Some research methodologies integrate RGB images with deep learning target detection algorithms for direct detection (Yu, R et al., 2021b; Wu et al., 2021);. Yu, R et al. utilized Faster R-CNN, YOLOv4, random forest, and support vector machine algorithms to detect early PWD. The target detection algorithm categorized PWD into four classes, with early PWD accuracy at 48.88%. Machine learning was employed for multi-classification tasks of PWD, achieving an overall classification accuracy of 75.33%. Wu, B et al. trained four deep learning models (Faster R-CNN ResNet50, Faster R-CNN ResNet101, YOLOv3 DarkNet53, and YOLOv3 MobileNet) for detection. Images ranging from 390 to 760nm were utilized for early PWD detection, with an average detection accuracy of 50.2%. While the target detection algorithm based on deep learning in RGB images is effective for mid- and late-stage PWD, its efficacy for early-stage PWD is moderate. This is primarily due to minimal changes in canopy color of early PWD diseased plants, leading to challenges in accurately identifying them using the deep learning-based target detection algorithm. In conclusion, reconstructing the spectral data from RGB images and extracting spectral information prove to be valuable and beneficial for the early detection of PWD.

### 5.3 The robustness of the model

**Figure 12:**
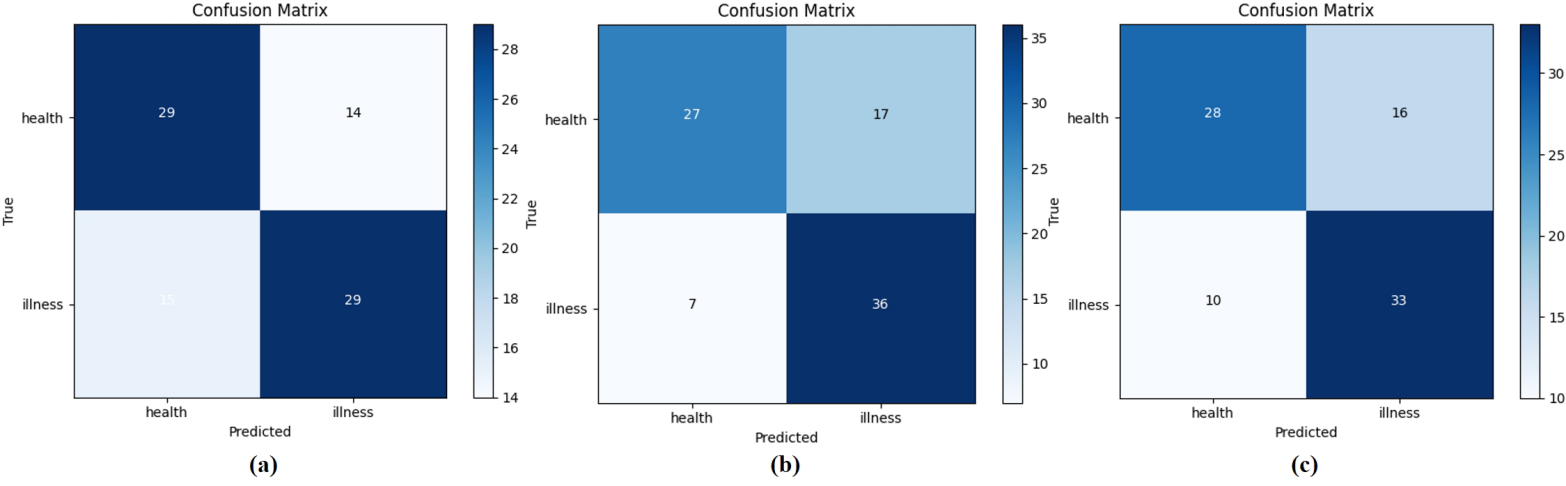
Samples from May, June, and August are selected as test sets respectively. **(A–C)** Respectively represent the confusion matrix of the test set on May 26th, the test set on June 8th, and the test set on August 17th.

This study randomly selected 87 samples in May, June, and August respectively as test sets to study the test results in different months. The confusion matrix of the test results was shown in **Figure 12**. Specifically, the samples in May were used as the test set, and the precision of the disease was 0.674, and the recall was 0.659; the samples in June were used as the test set, the precision of the disease was 0.68, and the recall was 0.84; Lastly, the samples in August were used as the test set, the precision of the disease was 0.67, recall was 0.77. The slight variation might stem from the sample discrepancies across various months, consequently influencing the classification outcomes. Moreover, the chi-square test conducted on 10 test results in this study revealed no significant statistical variance. Consequently, the hyperspectral image reconstruction network presented in this study, in conjunction with the SVM-based Early Stage PWD detection model, demonstrates robustness across samples from various months.

### 5.4 Conclusion

To summarize, In this paper, we proposed a method that utilizes UAV remote sensing color images, deep learning-based hyperspectral image reconstruction networks, and SVM classification to achieve early detection of pine wilt disease (PWD) in pine forests. The method achieved an accuracy of 77%. The key to achieving early detection of PWD in this study was the utilization of hyperspectral image reconstruction networks to reconstruct hyperspectral images The accuracy of the early detection method proposed in this study was comparable to or surpasses that of methods utilizing drone hyperspectral remote sensing technology. This article introduces a new hyperspectral image reconstruction network called DW3D, with the number of parameters being only 38% of MST++, the computational load is merely 71% of that in MST++, while achieving a relatively high-quality reconstruction. Hyperspectral remote sensing necessitates costly sensors for accurate data collection and entails time-consuming and complex data processing. In contrast, the method proposed in this paper reduces the detection cost, enhances data collection efficiency, and offers more advantages for large-area detection tasks. The establishment of the pine wilt disease (PWD) remote sensing dataset in this study serves as a foundation for early detection of PWD. As the dataset size grows in the future, the detection accuracy will also improve.

Current research encounters challenges in large-scale detection. The primary challenge involves integrating classification processing into reconstruction to establish an end-to-end detection model. The secondary challenge pertains to the limited richness of current data samples. Future endeavors should focus on enhancing early PWD samples to boost the detection model’s accuracy, facilitating more effective early PWD detection models in extensive agricultural regions.

## Data Availability

The raw data supporting the conclusions of this article will be made available by the authors, without undue reservation.
